# Conversion of Solar Energy into Electrical Energy Storage: Supercapacitor as an Ultrafast Energy‐Storage Device Made from Biodegradable Agar‐Agar as a Novel and Low‐Cost Carbon Precursor

**DOI:** 10.1002/gch2.201800037

**Published:** 2018-08-17

**Authors:** Manavalan Vijayakumar, Jyothirmayi Adduru, Tata Narasinga Rao, Mani Karthik

**Affiliations:** ^1^ Centre for Nanomaterials International Advanced Research Centre for Powder Metallurgy and New Materials (ARCI) Balapur, Hyderabad 500005 India

**Keywords:** agar‐agar, biomass‐derived carbon, energy conversion and storage, solar cells, supercapacitors

## Abstract

Solar cells hold promise as energy conversion devices but intermittent sunlight limits their continuous applications. The self‐powering integrated solar cells and electrical energy storage devices can be an alternative to resolve this problem. This study demonstrates the integration of solar cell with supercapacitor (SC) device and evaluates its performance for energy conversion and storage for practical validity. SC carbon is derived from agar‐agar as low‐cost carbon precursor and a high‐performance SC electrode is utilized for the first time. The fabricated SC shows an excellent specific capacitance of 170 F g^−1^ and retains 85% of its original value up to 15 000 charge/discharge cycles at 1 A g^−1^, and it holds a maximum energy density of 17.7 Wh kg^−1^. The integration of SCs (three cells in series with 5.4 V) with a commercial solar lantern for a self‐sustaining power pack is demonstrated. The SC is charged by solar cells in a few seconds and powers a solar lantern with 40 light‐emitting diodes without sunlight, demonstrates its potential for efficient conversion of solar energy into electrical energy storage. This result highlights that solar SC can be considered as an ultrafast next‐generation energy‐storage device that can mitigate the energy demand in the near future.

## 1 Introduction

Solar energy is considered as one of the most sustainable, abundant, unlimited, and clean energy resources to mankind, which can provide a greener path to fulfill the global energy demands of our modern society. The recent advanced renewable energy conversion and storage technologies have been led to continue growths in order to meet our future energy demands without any harmful emissions. Several technologies have already been developed and demonstrated on the efficient utilization tapping the solar energy which is further converted into green energy.^1^, ^2^, ^3^

Solar cells (photovoltaic, PV) for harvesting energy from sunlight have extensively been investigated and also commercialized because of high power‐conversion efficiency, low cost, and custom design.^4^, ^5^, ^6^ However, the output power from PV is fluctuating owing to the intermittent nature of the solar irradiation, which obstacles the practical incorporation of this technology for continuous applications. The self‐powering integrated solar cells and electrical‐energy‐storage devices could be an alternative to resolve this problem via simultaneous electric energy storage and manipulation of the output electric energy for continuous energy supply and utilized on demand.^1^, ^7^, ^8^ In order to address this, integration of the solar energy conversion and electrical energy storage into single compact devices with low weight, self‐powering, and efficient devices has already been made and demonstrated. It is also observed that the integration of solar cells with electrical‐energy‐storage unit not only realizes solar energy storage but also diminishes the fluctuation of solar irradiation as output power source.^1^, ^8^

Supercapacitors (SCs) and lithium batteries (LiBs) as electrical‐energy‐storage devices are extensively utilized not only for powering several portable electronic devices but also for plug‐in hybrid electric vehicles. Among these, SCs showed outstanding potential as compared with LiB because of their high power, long cycle life, and long‐term stability.^9^, ^10^, ^11^ SCs are commercially available but their widespread usage is restricted by their high cost and low energy density. These drawbacks can be mitigated by developing a new class of high‐performance carbon electrodes which consist of a combination of materials produced from abundant, cheap, and environmentally friendly resources with low processing costs.^12^ In this study, we introduce a new biomass‐based material that might have promising applications for SCs in the near future. On the other hand, electrolyte plays an important role in the SC performance and processing cost. SCs with aqueous electrolytes have received much research attention since they are safer, cheaper, and more environmentally friendly when compared with organic electrolytes.^13^, ^14^

In addition, aqueous SCs are inherently safe because no flammable or toxic liquids are used. This is very important when the SC is being connected directly on the backside of a solar panel, where the temperature becomes very high. The major limitation of aqueous SCs is their low voltage due to the possibility of gas evolution (hydrogen and/or oxygen) at higher potential. This implies a lower energy and power density for the aqueous electrolytes compared with the organic electrolytes. In order to achieve the desired voltage, more SCs with aqueous electrolytes would be needed.

The overpotential of hydrogen in neutral aqueous electrolytes such as Na_2_SO_4_ and Li_2_SO_4_ is higher than in either KOH or H_2_SO_4_ electrolytes as identified recently. A stable working potential window between 2.0 and 2.2 V can be achieved by using activated carbon (AC) based SC with 0.5 mol L^−1^ Na_2_SO_4_ in three‐electrode system, and this system also showed a long durability of more than 10 000 charge/discharge cycles.^13^, ^15^, ^16^ Consequently, even higher potential window of 2.2 V by using 1 mol L^−1^ Li_2_SO_4_ has been recently demonstrated and such a high potential window is attributed to dehydration energy of ions as well as high concentrations of local OH^−^ ions in the negative porous carbon electrode at neutral pH due to reduction of water molecule and concomitant chemisorptions of atomic hydrogen which do not favor hydrogen or oxygen evolution.^13^

In light of the above, in the present work, agar‐agar was used as a carbon source because of its low cost, easy availability, and biodegradable nature. Agar‐agar is a jelly‐like substance derived from polysaccharide agarose. Agar‐agar can be considered as a suitable carbon source for SC applications because it contains only polysaccharide that can be converted into pure carbon upon carbonization at 600 °C under N_2_ atmosphere. Generally, most of the biomass carbon sources are containing several elemental/mineral compositions, which are retained after carbonization even at high temperature up to 1000 °C and the final elemental composition like metals, metal oxides, and ashes should be removed by purification process that is generally expensive as well as has environmental threats.

**Figure 1:**
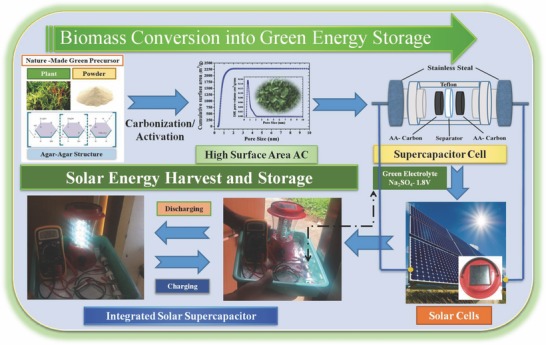
Schematic representation of biomass conversion into green energy storage.

The present research delineates fabrication of very high surface area (SA) AC (2250 m^2^ g^−1^) derived from biodegradable agar‐agar as a novel and economic carbon source and utilized as a high‐performance symmetric SC electrodes for the first time. The schematic representation of the overall concept of biomass conversion into green energy storage is shown in **Figure** **1**. The influence of electrode active mass per square centimeter on the specific capacitances, energy density, and power density was investigated, and the salient results are discussed. Interestingly, the fabricated SC cell showed a high specific capacitance of 170 F g^−1^ at 0.5 A g^−1^, excellent rate performance (70 F g^−1^ at 50 A g^−1^), and long‐term stability (15 000 cycles) in Na_2_SO_4_ as a green electrolyte. Moreover, we demonstrate a practical approach to integrate self‐sustaining power pack combining the symmetric SC with a commercial solar cell lantern (commercial solar lantern specification: 4 V, 1.6 Ah, 2–3 W). Importantly, the solar‐charged SC can power a commercial solar lantern with 40 light‐emitting diode (LED) lights in the absence of sunlight, which demonstrates its potential for efficient conversion of solar energy into electrical energy storage.

## 2 Results and Discussion

### 2.1 Textural and Morphology of the Carbon Electrode

**Figure 2:**
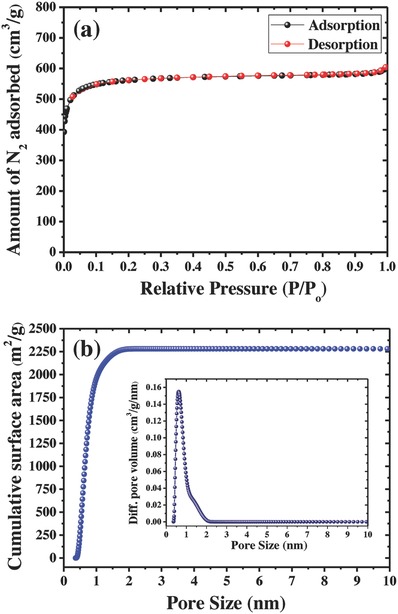
Textural properties of the AC: a) N_2_ adsorption–desorption isotherm and b) cumulative SA versus pore size distribution.

**Figure 3:**
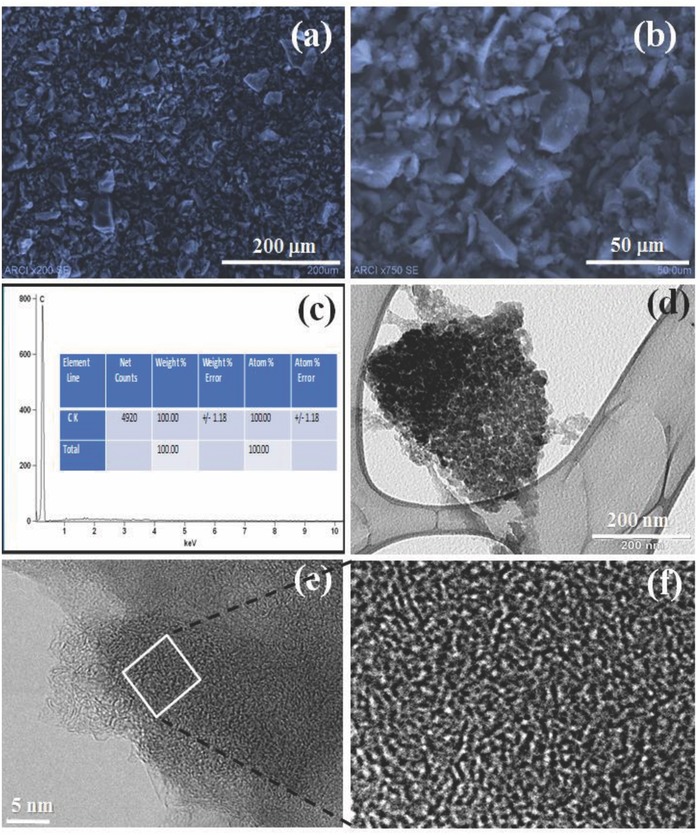
Surface morphology of the AC: a,b) SEM images, c) EDAX analysis, d,e) low and high magnifications of TEM image, and f) enlarge TEM image of selected area.

The textual properties of the AC were characterized by using N_2_ adsorption–desorption measurements. The Brunauer–Emmett–Teller (BET) isotherm as shown in **Figure** **2**a clearly exhibited a type I isotherm according to the IUPAC classification,^17^, ^18^, ^19^, ^20^ which reflects the microporous nature, and the isotherm at lower *P*/*P*_o_ values between 10^−7^ and 10^−5^ is corresponding to nitrogen filling in the micropore volume. It can be observed that no hysteresis loop was observed in isotherm branches, which indicates the saturation of isotherm. This feature indicates the presence of typical microporous materials. T‐plot results also showed that the micropore volumes of carbon are estimated to be 0.92 cm^3^ g^−1^. The specific SA of high microporous AC is calculated by using BET method, and it is around 2250 m^2^ g^−1^. The high specific surface of the obtained carbon electrode can offer sufficient electrode–electrolyte interaction to form electric double layers. The pore size distribution of the microporous AC obtained by 2‐dimensional non‐local density functional theory (2D NLDFT) is shown in Figure 2b (inset), which indicates the microspores in the range between 1.0 and 1.5 nm. The cumulative SA versus pore size of the AC is depicted in Figure 2b, demonstrating the exclusive presence of micropores without any major mesopores. The surface morphology of the AC samples was examined by using scanning electron microscopy (SEM) and transmission electron microscopy (TEM) as shown in **Figure** **3**. SEM pictures (Figure 3a,b) showed that the carbon particle size is in the range of 10–20 µm and TEM picture (Figure 3d–f) clearly indicates the uniform and highly disordered amorphous microporous structure of the carbon material. It can be observed from TEM images that the micropores of the AC are around 1.0–1.5 nm. This result is in good agreement with the pore size distributions obtained by 2D‐NLDFT. Furthermore, the purity of the carbon sample was determined by energy dispersive X‐ray analysis (EDAX) analysis as indicated in Figure 3c.

### 2.2 Electrochemical Performance

Generally, a typical commercial SC is composed of two carbon electrodes that are separated by a porous separator. In order to evaluate the practical electrochemical energy‐storage performance of agar‐agar‐derived carbon, two symmetric SC electrodes were fabricated by using 0.5 m Na_2_SO_4_ as a green electrolyte. This present study mainly emphasizes the effects of active mass of the electrodes on the electrochemical performances and the fluctuation of specific capacitance as well as energy and power density with respect of active mass of the electrodes were studied and elaborated in the following sections.

#### 2.2.1 Influences of Active Mass of the Electrodes

The active mass of the electrodes is considered as major influence parameters on the electrochemical performances.^21^ In order to construct the SC with an optimize energy density or power density, the SC electrode are made with the thickness of about 100–200 µm.^21^ The less active mass of the electrode or thin electrode can always lead to an overstatement of a material's performance because, on the one hand, the significant errors could be possible during the measurement of active mass of the electrode when handling and weighing the microgram‐sized electrodes, and on the other hand, the signal‐to‐noise ratio is also the major concern. For reliable electrochemical measurements, a SC electrode should have thickness and active mass similar to the commercial electrodes, i.e., 100–200 µm and 10–12 mg cm^−2^, respectively.^21^, ^22^, ^23^, ^24^ In view of the above, various active masses of the electrodes were prepared and investigated the electrochemical performances.

**Figure 4:**
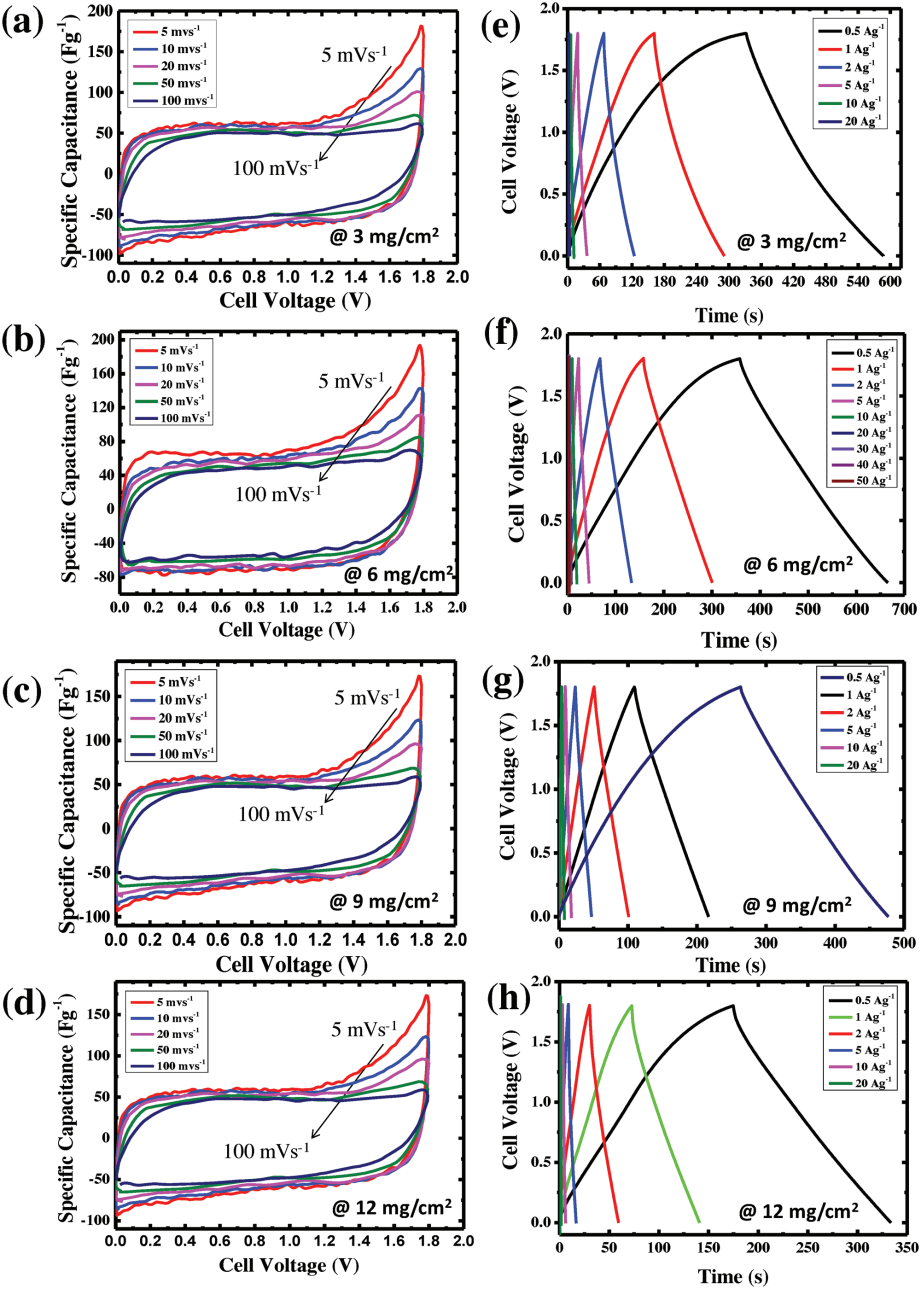
Electrochemical performance of biomass‐derived carbon with two‐electrode system using 0.5 m Na_2_SO_4_ as electrolyte at different mass loadings. a–d) CV curve at 20 mV s^−1^ scan rate and e–h) GCD curves at 1 A g^−1^ current density.

Initially, the electrochemical properties and performances of a symmetric SC cells were evaluated by cyclic voltammetry (CV) measurements with a potential window of 0–1.8 V at different scan rates. In order to optimize the performance of the active mass of the electrode per cm^2^, CV performance on different active mass loadings of the electrode such as 3, 6, 9, and 12 mg cm^−2^ were investigated and the results are shown in **Figure** **4**a–d. It can be observed from Figure 4a–d, all the symmetric electrodes showed a typical rectangular CV curve at a lower scan rate. On the other hand, CV loop exhibits a distortion rectangular shape due to ohmic resistance at a higher scan rate. It is well known that cyclic voltammogram with broader area exhibits higher capacitance. From the results, it can be observed that CV curve for electrode active mass about 6 mg cm^−2^ displays a broader rectangular shape without significant distortion which corresponds to an ideal electrochemical double layer capacitive behavior. In addition, the rectangular shapes are also well retained even at 100 mV S^−1^, which indicates a rapid *I*–*V* response and fast charge–discharge characteristic of the SC at higher scan rate. In addition, it exhibited a higher capacitance and a better reversibility even at higher scan rate compared with other electrode samples, suggesting the electrode with optimum active mass loading (6 mg cm^−2^) exhibits an excellent rate capability. A similar observation was reported by Hu et al.^23^ and highlighted that it is very important to use appropriate electrode thickness and active mass of the electrode for meaningful values of specific capacitance and energy density for practical applications. It is worthy to mention here that the active mass loading of the commercial electrode is around 10 mg cm^−2^ as indicated earlier but the optimum active mass loading of the electrode in this study was found to be 6 mg cm^−2^ because AC has a very high SA (2250 m^2^ g^−1^) with low density and hence the thickness of the electrode is around ≈90 µm when the carbon material is reached around 6 mg cm^−2^, it is very close to commercial electrode (≈110 µm). When the active mass loading increased from 9 to 12 mg cm^−2^, the thickness of the electrodes is also increased from ≈140 to ≈180 µm.

**Figure 5:**
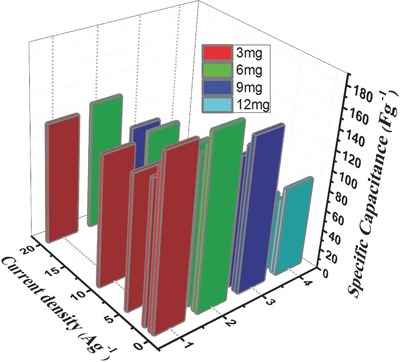
Effect of mass loading of the electrodes on specific capacitance as a function of current density.

The galvanostatic charge–discharge (GCD) profiles of all the electrodes with various current densities are depicted in Figure 4e–h. The charge–discharge curves of all the electrodes are highly symmetrical and triangular curve with small voltage drop (equivalent series resistance (ESR)) suggesting good SC performance and suggesting that both carbon electrodes have excellent reversibility as well as Coulombic efficiency. The current density versus specific capacitance of the four different mass loading electrodes is depicted in **Figure** **5**. For the same electrolyte, when the active mass loading of the electrode is increased from 6 to 12 mg cm^−2^, the specific capacitance was decreased from 170 to 88 F g^−1^ (around 48% decreased) at current density of 0.5 A g^−1^. This effect was even more pronounced at a higher current density. The specific capacitance was drastically decreased from 126 to 34 F g^−1^ (around 73% decreased), when the active mass loading is increased from 6 to 12 mg cm^−2^. From the results, it was found that the electrode with 6 mg cm^−2^ showed a higher discharge time consequently; it has higher specific capacitance as compared with other electrode samples. In addition, it also exhibited good capacitance retention about 70 F g^−1^ even at 50 A g^−1^, demonstrating the excellent reversibility and less internal resistance. From the CV and GCD measurements, it can be concluded that electrode with 6 mg cm^−2^ is an optimum mass and showed a higher supercapacitive performance. The obtained results clearly indicated that the active mass loading of the electrode is major influencing factor that severely affects the SC performance.^24^ It is very important to use appropriate electrode thicknesses and masses for any meaning to be attached to reported values of specific capacitance and energy density.

**Figure 6:**
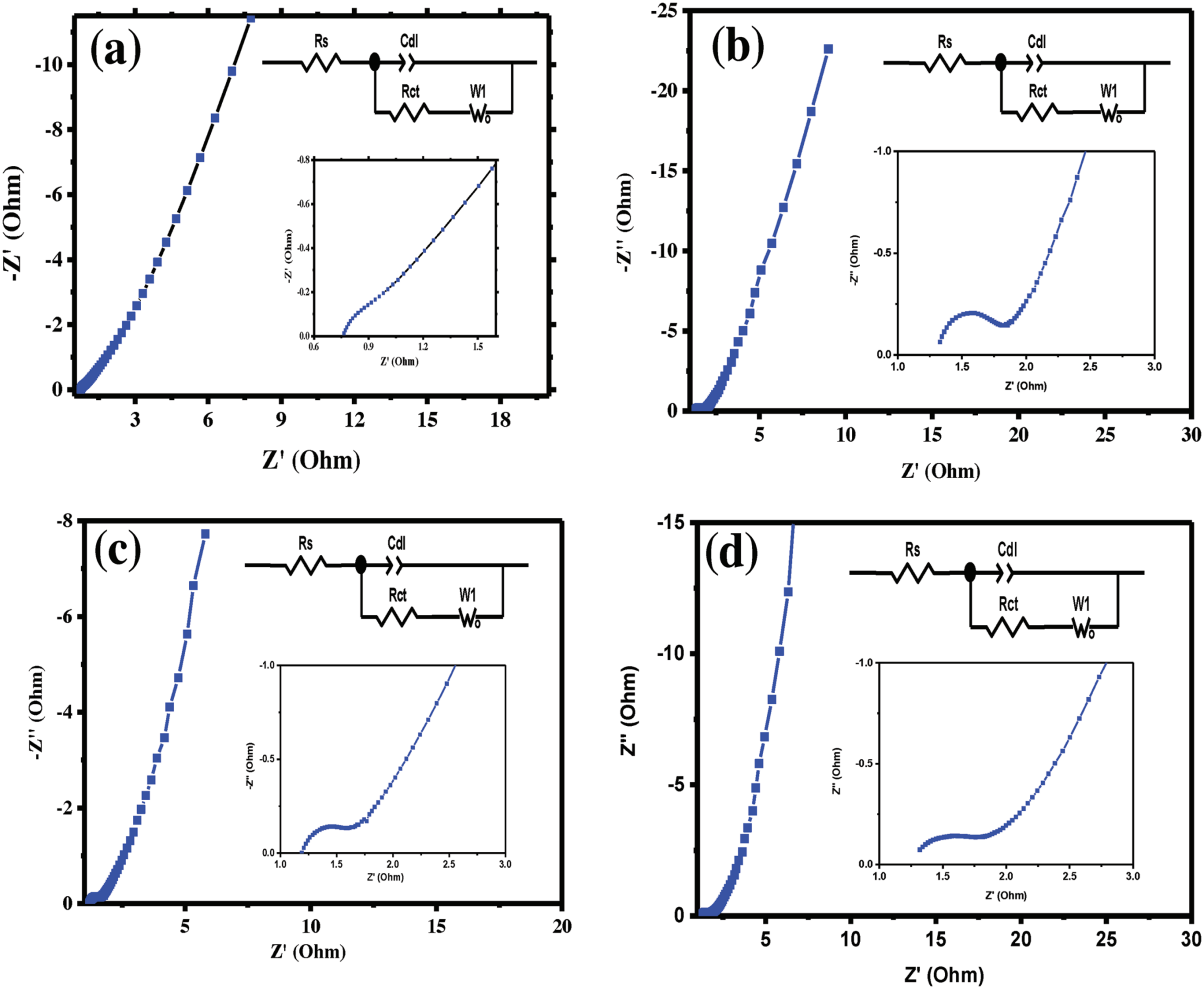
Comparison of Nyquist plots for symmetric SC cells using different electrode masses in 0.5 m Na_2_SO_4_ electrolyte.

In order to test the series resistance and charge transfer resistance of the SC electrodes, electrochemical impedance spectroscopy (EIS) was used. The SC electrodes with low resistance are electrochemically preferred for better commercial device applications. In this concern, all the SC cells were investigated by EIS. Generally, a SC behaves like a pure resistor at high frequencies, besides at relatively low frequencies, it behaves like a capacitor. It is well accepted that the diameter of the semicircle at high‐frequency region reflects the polarization resistance or charge transfer resistance (*R*_ct_), and the vertical line at low frequency indicates ideal capacitor behavior and low diffusion resistance of electrolyte ions.^24^ The Nyquist plots of the different electrode samples in 0.5 m Na_2_SO_4_ electrolyte are illustrated in **Figure** **6**, indicating the impedances in the frequency range between 0.01 Hz and 100 kHz. Nyquist plot shows a vertical straight line perpendicular to the horizontal coordinate for an ideal porous electrode. However, Nyquist plot can be divided into three parts for real porous electrodes, i.e., high‐, medium‐, and low‐frequency regions. Nyquist plot shows semicircle, and the real axis intercept is the ESR at high‐frequency region.^25^ The charge transfer resistance of the electrode materials can be represented via width of the semicircle impedance loop. In addition, same fabrication assembly and electrolyte were used for all the four electrodes and hence the electrolyte resistance and contact resistance of the electrodes seem to be identical. However, it shows different values with respect to the active mass of the electrode, i.e., 0.25 Ω at 3 mg cm^−2^, 0.34 Ω at 6 mg cm^−2^, 0.40 Ω at 9 mg cm^−2^, and 0.56 Ω at 12 mg cm^−2^. The Nyquist plot showed straight line for an EDLC at low‐frequency region. Generally, the more ideal capacitor shows more vertical line. The obtained results showed that all the SC electrodes have an almost vertical line, which indicates ideal SC behavior. As shown in Figure 6, the length of the line with a slope of 45° at 6 mg cm^−2^ is much shorter than that of other electrode samples, indicating a lower diffusion resistance in the electrode with 6 mg cm^−2^. Consequently, electrode at 6 mg cm^−2^ represents more ideal Nyquist curve than other electrodes. This result can be attributed to more favorable access of ions in the electrolyte with electrode in optimum active mass loading with optimum electrode thickness. The electrochemical impedance results clearly demonstrated that the optimum active mass electrode at 6 mg cm^−2^ showed better SC performance. Because the optimum level of electrode thickness and active mass loading of the electrode favors the electric double layer formation effectively it can store more charge on the porous carbon electrode surface.

**Figure 7:**
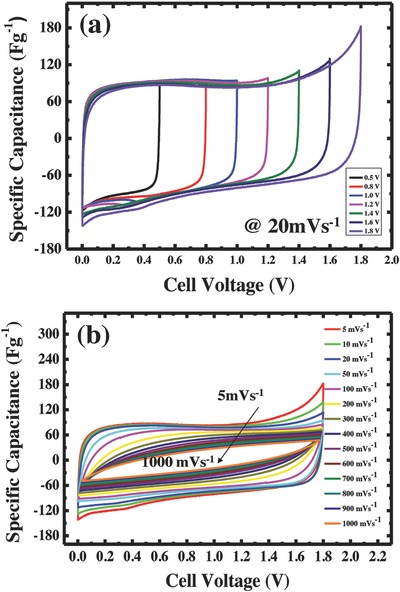
Electrochemical performance of SC electrode with optimized mass loading: a) CV curves with different voltage windows, b) CV curves with different scan rates.

Furthermore, the electrochemical stability of the electrolyte and rate capability of the optimised SC electrode were further investigated. In order to assess the stability of the electrolyte, various potential windows from 0 to 1.8 V with a scan rate of 5 mV s^−1^ were used for CV as shown in **Figure** **7**a. From CV curves, it can be observed that typical rectangular shape was obtained that indicates a high specific capacitance without any electrolyte decomposition even in several cycles. In addition, the high rate capability of the electrode at 1.8 V with different sweep rates between 5 and 1000 mV s^−1^ was investigated and the obtained CV curves are depicted in Figure 7b. As the scan rate increases, the current density is proportional to the scan rate, which is a typical result of adsorption and desorption characteristics of the EDLCs. In addition, the CV curves maintain a relatively rectangular shape with slight distortion up to 200 mV s^−1^ indicating an excellent capacitance behavior and fast diffusion of electrolyte ions into the electrode. When increasing the scan rate further, the rectangular to distortion ratio is also increased due to the internal resistance arising during the fast diffusion of ions into the electrode.

**Figure 8:**
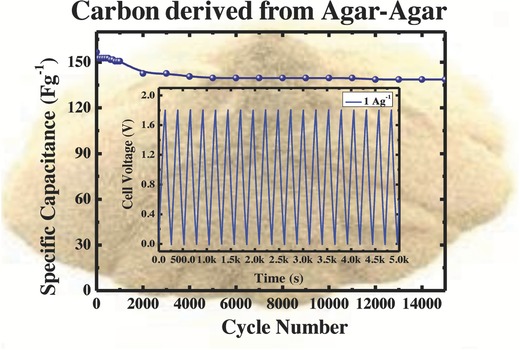
Cyclic performance of symmetric SC cell (inset: charge–discharge profile at 1 A g^−1^ current density).

From practical point of view, the stability of SCs upon charge–discharge cycling is another important aspect. The electrode stability was investigated by long‐term charge–discharge cycle at current density of 1 A g^−1^ with a potential window of 0 to 1.8 V as shown in **Figure** **8**. It was observed that after 15 000 cycles, the system still retains 85% of its initial capacitance with less ESR, evidencing the superb robustness of the carbon electrode, at high discharge rates.

**Figure 9:**
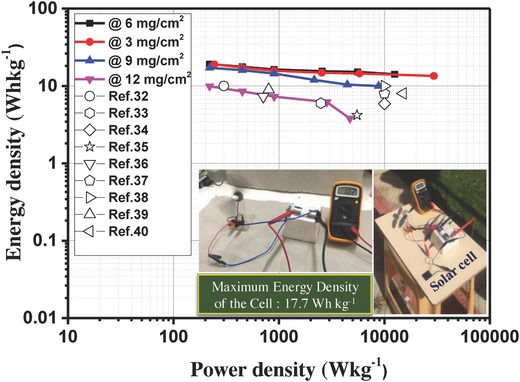
Typical Ragone plot of the SC cell in 0.5 m Na_2_SO_4_ electrolyte with different mass loadings of the electrode compared with other biomass‐carbon based SC reported in the literature (inset: demonstration of the SC cells).

For practical applications, the energy density and power density of the SC device are the major parameters and are represented via Ragone plot. Hence, Ragone plot of the symmetric SC cell is shown in **Figure** **9**, which indicates that the SC cell has promising power––energy characteristics. The SC cell with 0.5 m Na_2_SO_4_ electrolyte shows the highest energy density of around 17.7 Wh kg^−1^ with the corresponding power density of around 450 W kg^−1^ at current density of 1 A g^−1^, which showed higher value as compared with other biomass‐carbon‐based SC electrodes reported in the literature.^26^, ^27^, ^28^, ^29^, ^30^, ^31^, ^32^, ^33^, ^34^, ^35^, ^36^, ^37^, ^38^, ^39^ For example, the obtained energy density value in this study is comparable to those previously reported for other carbon‐based electrodes, such as petroleum coke‐based carbon (≈8 Wh kg^−1^),^26^, ^31^ activated porous carbons (≈5‐10 Wh kg^−1^),^27^, ^32^, ^33^ porous carbon shell (≈10 Wh kg^−1^),^28^ rod‐shaped porous carbon (≈17–20 Wh kg^−1^),^29^ and nitrogen‐doped porous carbon (≈9–20 Wh kg^−1^).^30^, ^38^, ^39^ Furthermore, the power density of the SC can reach as high as 14 kW kg^−1^ when the energy density is still maintained about 10.5 Wh kg^−1^ at 30 A g^−1^, confirming the excellent rate capability performance of the SC.

### 2.3 Demonstration of Supercapacitor Device

**Figure 10:**
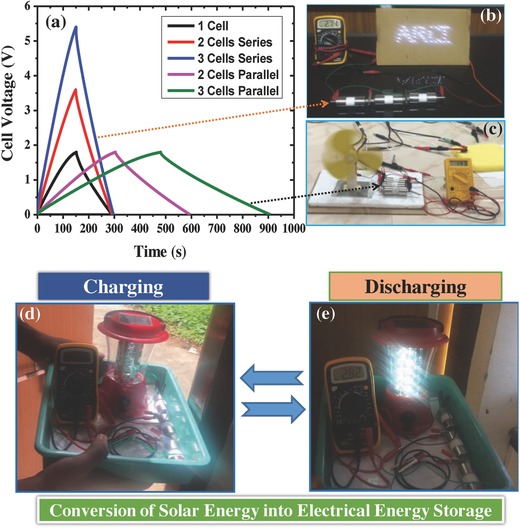
Demonstration of SC device by using biomass‐derived carbon as an effective symmetric SC electrode. a) Charge–discharge profile for series and parallel combination of SC cells, b) three cells in series connection, c) three cells in parallel connection, d) SC cells charged by solar cell, e) SC‐powered solar lantern.

In the practical application, the fabricated AC‐based SC cells were further tested by using mini‐proto‐type devices as depicted in **Figure** **10**. In order to increase the capacitance and voltage, a set of the SC cells were connected in the series and parallel, and they were tested. When three SC cells are connected in parallel, the total capacitance is increased as demonstrated by running the fan with 120 mA of discharge current (Figure 10c). If the SC cells are connected in series, the total voltage is increased as demonstrated by illuminating 40 LED lights (arranged in the form of ARCI alphabets) and solar lantern (Figure 10d,e). A practical approach to combine the symmetric SC (three cells in series with 5.4 V) with a commercial solar cells used in the lantern was developed to integrate self‐sustaining power pack as shown in Figure 10d,e. Importantly, the solar‐charged SC can power a commercial solar lantern with 40 LED lights in the absence of sunlight, which demonstrates its potential for efficient conversion of solar energy into the electrical energy storage. The efficient capture and conversion of the sun energy into electrical energy and then storage in the device has showed the utilization of renewable energy and showed to produce the economic electricity. These results clearly demonstrate that the solar cells integrated with SC can consider as an ultrafast next‐generation energy‐storage device that can mitigate the energy demand and reduce the cost of the electricity in the near future.

## 3 Conclusions

A high‐performance symmetric SC electrode was fabricated by using high SA AC obtained from biodegradable agar‐agar as an economic, efficient, and green carbon precursor. The structural and surface morphological investigation showed that the obtained AC exhibited an amorphous nature with very high SA and narrow pore size distribution. The electrochemical properties of the symmetric SCs were studied by utilizing green aqueous neutral electrolyte Na_2_SO_4_ due to its operating potential window 1.8 V, and it has almost double the potential window as compared with other aqueous electrolytes such as KOH and H_2_SO_4_. Interestingly, the fabricated symmetric SCs showed an excellent specific capacitance of 170 F g^−1^ at 0.5 A g^−1^ and excellent cyclic stability especially with high capacitance retention around 85% even after 15 000 charge–discharge cycles, and it also shows a high energy density of 17.7 Wh kg^−1^ with the corresponding power density of 450 W kg^−1^ at 1 A g^−1^, which exhibit higher value as compared with other carbon based SC electrodes reported in the literature. This study clearly highlighted that the active mass loading and thickness of electrode are crucial factors that influence the SC performance of the electrode. These results demonstrate the possibility of the integration and hybridizing energy conversion and energy storage and the solar cells integrated with SC as a next‐generation clean energy‐storage device can mitigate the energy demand and reduce the cost of the electricity in the near future.

## 4 Experimental Section

*Fabrication of Activated Porous Carbon*: The high SA carbon from agar‐agar was obtained by precarbonization followed by chemical activation. Briefly, the raw agar‐agar powder was precarbonized by using a tubular furnace under N_2_ flow at 600 °C for 1 h with heating rate of 5 °C min^−1^ and the carbon yield from the precarbonization of agar‐agar was around 25 wt%. The resulted carbon powder was mixed with KOH (weight ratio of carbon and KOH is 1:3). Then, the above mixture was placed in the alumina crucible and kept in the stainless steel tube within a tubular furnace. The activation of carbon sample was carried out at 850 °C for 1 h with a rate of heating 5 °C min^−1^ under N_2_ flow. The resulted AC sample was stirred in 1 m HCl for 1 h and washed thoroughly with double distilled water up to neutral pH. Finally, the carbon sample was dried for 12 h at 80 °C.

*Characterization of the Materials*: The surface morphologies of the materials were analyzed by using SEM (SEM‐Hitachi SU‐70, Japan) and TEM (Model: FEI Tecnai G‐20200 K eV). The elemental mapping and composition of the activated samples were analyzed by using EDAX. The SA, pore diameter, and pore volume of the carbon samples were characterized by N_2_ adsorption–desorption isotherms at 77 K using a Micromeritics, ASAP 2020 SA analyzer. Prior to the measurements, the carbon samples were degassed under vacuum (10^−3^ mbar) at 350 °C for 8 h. The pore size distributions were obtained by utilizing 2D‐NLDFT as reported in the previous report.^17^, ^18^

*Electrodes Preparation and Electrochemical Measurements*: The symmetric carbon–carbon electrodes were fabricated by mixer of 90 wt% of active material (AC) and 10 wt% of binder (polyvinylidene fluoride (PVDF)). The mixture was homogenized by adding a few drops of *N*‐methyl‐2‐pyrolidone, was made into a uniform slurry, and was then worked out until plasticity was achieved. The slurry was then coated onto nickel‐mesh sheet with 12 mm in diameter with a thickness of ≈200 µm, and the electrodes were dried at 120 °C for overnight. The disk‐shaped identical two electrodes of 12 mm in diameter were prepared with various thicknesses, and the final electrodes were separately weighed. Then, the thickness of the electrodes was measured. Finally, carbon–carbon symmetric SC cell was fabricated by using two identical carbon electrodes, a porous membrane separator (glass fiber), and two stainless steel current collectors assembled in a Teflon Swagelok cell. 0.5 m Na_2_SO_4_ was used as a green electrolyte.

The electrochemical performances such as charge–discharge, CV, and electrochemical impedance were recorded by using potential static electrochemical work station Solartron SI‐1287 and Princeton Applied Research – Ametek with a two‐electrode configuration at different current densities.

Specific capacitance is the capacitance per unit mass for one electrode. The specific capacitance of the SC electrodes was calculated from the discharge curves by using Equation (1), 23

(1) $$C_{\mathrm{sp}}\left(F\;g^{-1}\right)=4*C\;m^{-1}$$

Cell capacitance was measured from galvanostatic or constant current discharge curves using Equation (2), 23

(2) $$C=I/\left(dV\mathrm{/d}t\right)$$

where *C*_sp_ is the specific capacitance of the electrode, *C* is the measured capacitance for the two‐electrode cell, *m* (g) is the active mass of the both electrodes, *I* (A) is the discharge current, d*V* (V) is the total potential window, and d*t* (s) – the discharge time, respectively.

The energy and power densities of the two symmetrical SC electrodes were calculated by using Equations (3) and (4):

(3) $$E\left(\mathrm{Wh}\;{\mathrm{kg}}^{-1}\right)=1/2\;C_{\mathrm{sp}}V^{2}$$

(4) $$P(W\;{\mathrm{kg}}^{-1})=\frac{E}{\Delta t}$$

where, *C*_sp_ is the specific capacitance of the symmetrical SC (F g^−1^) calculated from Equation (1), *V* (V) refers to the potential change of galvanostatic discharge process, Δ*t* (s) is discharge time.

## Conflict of Interest

The authors declare no conflict of interest.
